# Compensatory combination of mTOR and TrxR inhibitors to cause oxidative stress and regression of tumors

**DOI:** 10.7150/thno.52077

**Published:** 2021-02-25

**Authors:** Yiqun Xia, Jundixia Chen, Yun Yu, Fengjiao Wu, Xin Shen, Chenyu Qiu, Tingting Zhang, Lin Hong, Peisen Zheng, Rongrong Shao, Chenxin Xu, Fang Wu, Wei Chen, Congying Xie, Ri Cui, Peng Zou

**Affiliations:** 1 The First Affiliated Hospital of Wenzhou Medical University, Wenzhou Medical University, Wenzhou 325035, China.; 2 Cancer and Anticancer Drug Research Center, School of Pharmaceutical Sciences, Wenzhou Medical University, Wenzhou 325035, China.; 3 Biomedical Collaborative Innovation Center of Zhejiang Province, Wenzhou University, Wenzhou 325035, China.; 4 Wenzhou University-Wenzhou Medical University Collaborative Innovation Center of Biomedical, Wenzhou 325035, China.

**Keywords:** mTOR, thioredoxin reductase, oxidative stress, autophagy, c-Jun N-terminal Kinase

## Abstract

**Background:** Cancer is a leading cause of death worldwide. Extensive research over decades has led to the development of therapies that inhibit oncogenic signaling pathways. The mammalian target of rapamycin (mTOR) signaling pathway plays an important role in the development of many cancers. Several mTOR inhibitors are approved for the treatment of cancers. However, the anticancer efficacies of mTOR inhibitor monotherapy are still limited.

**Methods:** Western blot was used to detect the expression of indicated molecules. Thioredoxin reductase (TrxR) activity in cells was determined by the endpoint insulin reduction assay. Immunofluorescence staining was used to analyze precise location and expression of target proteins. Nude mice were used for xenograft tumor models.

**Results:** We identified a synergistic lethal interaction of mTOR and TrxR inhibitors and elucidated the underlying molecular mechanisms of this synergism. We demonstrated that mTOR and TrxR inhibitors cooperated to induce cell death by triggering oxidative stress, which led to activation of autophagy, endoplasmic reticulum (ER) stress and c-Jun N-terminal Kinase (JNK) signaling pathway in cancer cells. Remarkably, we found that auranofin (AF) combined with everolimus significantly suppressed tumor growth in HCT116 and SGC-7901 xenograft models with no significant signs of toxicity.

**Conclusion:** Our findings identify a promising therapeutic combination for cancer and has important implications for developing mTOR inhibitor-based combination treatments.

## Introduction

Cells need to tightly regulate their cellular energetics, metabolism and redox balance to achieve and maintain proper growth and proliferation. In cancer, the well-controlled physiological homeostasis was destroyed due to deregulation of multiple signaling pathways. Altered signal transduction during tumor formation and progression often provides tumor cells with growth and survival advantages 1. However, the alterations in cell signaling and metabolism also create therapeutic vulnerabilities in cancer cells 2, 3. Thus, understanding the mechanisms responsible for cellular adaptation may lead to the development of successful strategies for molecular-targeted medicine and identification of synthetic lethal partners.

Reactive oxygen species (ROS) are continually generated and eliminated in biological systems, and plays a critical role in various physiological and pathological conditions. Under normal physiological conditions, cells aim to maintain redox homeostasis with a low level of basal ROS by controlling the balance between oxidants and antioxidants exists. In contrast, most cancer cells exhibit increased generation of ROS 4-6. The increase in ROS production is associated with abnormal cell growth and reflects a disruption of redox homeostasis in cancer cells 7. However, as excessive generation of ROS can also be toxic to the cells, cancer cells with increased oxidative stress are likely to be more sensitive to agents that increase ROS generation 8, 9. Therefore, it might be possible to selectively kill cancer cells by further ROS insults induced by exogenous agents without causing significant toxicity to normal cells.

Thioredoxin reductase (TrxR) is a selenoprotein that functions to reduce the oxidoreductase thioredoxin (Trx) in a NADPH dependent manner, and plays an important role in regulating the cellular redox balance 10. Since the expression of TrxR is up-regulated in a variety of human tumors and associated with increased tumor growth, drug resistance, and poor patient prognosis, TrxR has emerged as a promising target for cancer therapy 11, 12. Thus, recent years have witnessed increasing attention to develop novel inhibitors of TrxR as potential antitumor agents 13-15. In our previous study, we found that TrxR expression and activity were markedly enhanced in gastric cancer tissues, and identified TrxR as a target of WZ26 and piperlongumine 16, 17. These observations indicate that TrxR is a promising target for the treatment of cancer.

The mammalian target of rapamycin (mTOR) kinase is a master regulator of protein synthesis and cell growth 18. Cancer cells are often addicted to the higher mTOR signaling network due to its critical role in integrating extracellular cues and intracellular signaling 19, 20. The critical role of mTOR in cancer cell biology has stimulated interest in mTOR inhibitors 21. At present, mTOR inhibitors have been extensively studied and approved by the Food and Drug Administration (FDA) to treat advanced renal cell carcinoma 22. However, mTOR-targeted cancer therapies still have significant challenges because of the anticancer efficacies of mTOR inhibitor monotherapy are limited in clinical treatment 23-25.

In addition to its well-established role in protein synthesis and cell growth, mTOR also regulates the production of reduced form of glutathione. Accordingly, mTOR inhibitors have been shown to increase ROS levels in cancer cells. Nevertheless, mTOR inhibitors exert only cytostatic effects in these cancer cells 26, 27. We therefore sought to identify other targeted agents that might cooperate with mTOR inhibitors to enhance oxidative stress beyond threshold levels, thereby more effectively killing cancer cells. Here, we identify a promising drug combination for cancer and elucidate the underlying molecular mechanisms of this synergism. We show that mTOR and TrxR inhibitors synergize to induce cancer cell death by triggering oxidative stress. Importantly, this drug combination significantly suppresses tumor growth in HCT116 and SGC-7901 xenograft models. Together, our findings identify a promising new therapeutic combination for cancer and have important implications for the development of molecular-targeted therapies.

## Methods

### Materials

Auranofin (AF), AZD8055 and 3-Methyladenine (3-MA) were purchased from Targetmol (Boston, USA). JNK inhibitor SP600125 was obtained from Selleck Chemicals (Houston, USA). Rapamycin (Rap), everolimus (Eve) and N-acetyl-L-cysteine (NAC) were purchased from Aladdin Industrial Corporation (Shanghai, China). Antibodies of p-JNK, JNK, ATF4, p-eIF2α and eIF2α were purchased from Cell Signaling Technology (Danvers, USA). Antibodies against TrxR1 and GAPDH were purchased from Santa Cruz Biotechnology (Santa Cruz, USA). Antibody of CHOP/DDIT3 and Nrf2 were purchased from Abcam (Cambridge, USA). Antibody of LC3 was purchased from Proteintech (Rosemont, USA). The 53BP1 antibody was purchased from Novus Biologicals (Littleton, CO, USA).

### Cell culture

HCT116, RKO, SGC-7901, AGS and NRK-52E cell lines were obtained from the Cell Bank of Shanghai Institute of Biochemistry and Cell Biology, Chinese Academy of Sciences. HCT116 cells were grown in McCoy's 5A medium plus 10% fetal bovine serum (FBS). RKO cells were grown in minimun essential medium plus 10% FBS. SGC-7901 and AGS cells were grown in RPMI 1640 medium plus 10% FBS. NRK-52E cells were grown in DMEM plus 10% FBS. Mouse peritoneal macrophage (MPM) cells were obtained as previously described 28. All the cells were cultured in a humidified incubator with 5% CO_2_ at 37 °C.

### Cell viability assay

Approximately 8,000 cells per well were seeded in 96-well plates and incubated overnight. Next, the cells were treated with the test compounds for 24 h. Cell viability was measured using a methyl thiazolyl tetrazolium assay. The drug interaction was evaluated by using the combination index (CI) according to the Chou-Talalay method 29.

### Measurement of intracellular ROS

The fluorescent probe 2, 7-dichlorofluorescein diacetate (DCFH-DA) was employed to detect intracellular ROS levels. Briefly, cells were plated in 6-well plates and incubated overnight. Cells were treated with the test compounds for the indicated times. Next, the cells were stained with 10 μM DCFH-DA for 30 min before collecting. For quantitative assessment of intracellular ROS levels, the cells were collected and analyzed by FACSCalibur flow cytometer.

### Western blot analysis

Cells were seeded in 6-well plates and incubated overnight. After various treatments, the cells were washed once with 1 ml of phosphate-buffered saline and lysed using cell lysis buffer. The same amount of lysate proteins were separated by 10% SDS-PAGE and electroblotted onto PVDF transfer membranes. The blots were blocked with five percent non-fat milk in TBST for 2 h at room temperature. Then incubated with specific primary antibodies overnight at 4 °C. HRP-conjugated secondary antibodies and ECL substrate (Bio-Rad, Hercules, CA) were used for detection.

### Tandem mRFP-GFP fluorescence microscopy

A tandem monomeric RFP-GFP-tagged LC3 lentivirus (GeneChem, Shanghai, China) was used to detect autophagy flux. Briefly, cells were seeded in 12-well plates and incubated overnight. Next, the cells were infected lentivirus for 72 h according to the manufacturer's protocol. For evaluating tandem fluorescent LC3 puncta, the infected cells were treated with the test compounds for the indicated times. The images were obtained using a Leica fluorescence microscope.

### Measurement of TrxR activity

Cells were seeded in 6-well plates and incubated overnight. Next, the cells were treated with the test compounds for the indicated times and lysed with lysis buffer. TrxR activity in cell lysates was measured using an endpoint insulin reduction assay as previously described 17.

### Immunofluorescence staining

Cells were seeded on sterile cover glasses placed in the 6-well plates and incubated overnight. Next, the cells were treated with the test compounds for 20 h. For immunofluorescence, the cells were stained with a primary antibody (53BP1, 1:2,000 dilution) overnight at 4 °C. Next, the cells were incubated with a DyLight 488 conjugated secondary antibody for 1.5 h at room temperature. The images were obtained using a Leica fluorescence microscope.

### Xenograft experiments

Five-week-old athymic BALB/c nude mice (total n = 34) were used for *in vivo* experiments. All animals used in this study were handled according to the Institutional Animal Care and Use Committee (IACUC) guidelines, Wenzhou Medical University. The animals were housed at a constant room temperature with a 12 h light/12 h dark cycle and fed a standard rodent diet and water. HCT116 cells (5 × 10^6^ cells in 100 μl of phosphate-buffered saline) were injected subcutaneously into the flank of nude mice. Next, the mice were divided into four experimental groups on randomization and blinding, with six mice in each group. The mice were treated with auranofin (3 mg/kg), everolimus (5 mg/kg), or the combination by intraperitoneal (i.p.) injection once every other day at the indicated doses. Similarly, SGC-7901 cells (5 × 10^6^ cells in 100 μl of phosphate-buffered saline) were injected subcutaneously into the flank of nude mice. Next, the mice were divided into two experimental groups on randomization and blinding, with five mice in each group. The mice were treated with the combination of auranofin (3 mg/kg) and everolimus (5 mg/kg) by intraperitoneal (i.p.) injection once every other day. The tumor volumes were measured to observe dynamic changes in tumor growth and calculated according to the formula: V (mm^3^) = 0.5 × D × d^2^, where D and d are the longest and the shortest diameters, respectively. At the end of the experiment, all nude mice were sacrificed, and the tumor tissues were removed and measured.

### MDA assay

Malondialdehyde (MDA) is a terminal product of lipid peroxidation. For the MDA assay, tissue proteins of tumor xenograft were homogenized in ice-cold RIPA buffer. The protein concentrations were determined using the Bradford assay (Bio-Rad, Hercules, CA). The MDA levels were detected according to the protocol described previously 17.

### Statistical analysis

The data are expressed as means ± standard error of the mean (SEM). Significant differences between control and experimental groups were determined by t-test analyses using statistical software, GraphPad Prism 5.0. A probability (P) value of < 0.05 was considered statistically significant.

## Results

### mTOR and TrxR inhibitors synergize to induce cell death in gastric and colon cancer cells

**Figure 1:**
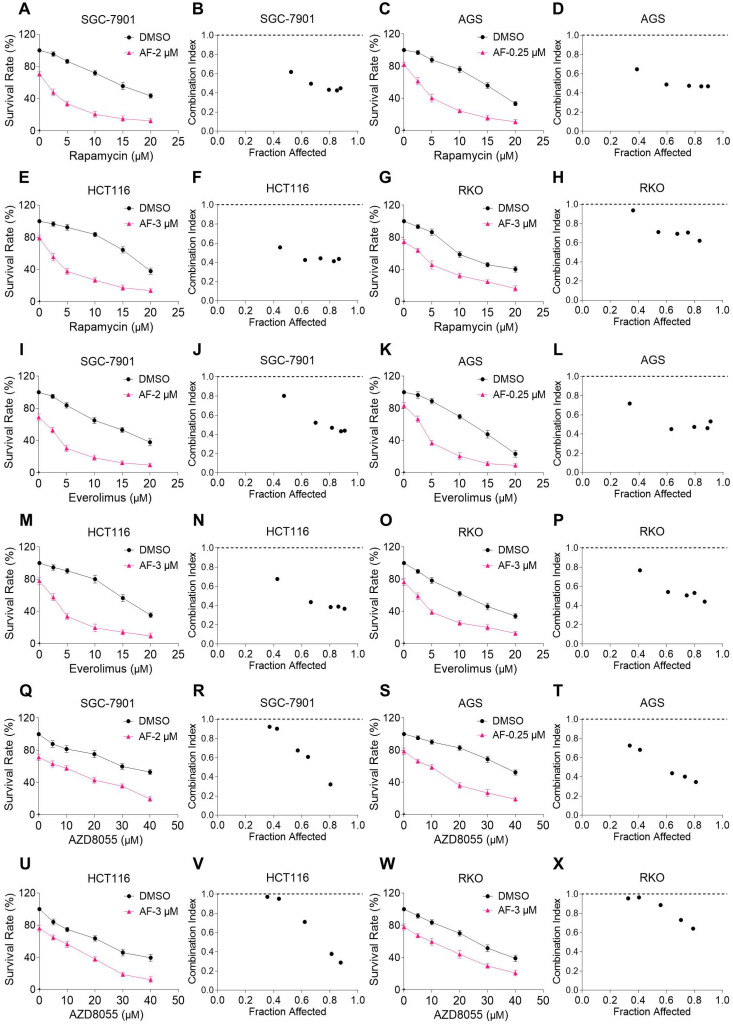
** mTOR and TrxR inhibitors synergize to induce cell death in gastric and colon cancer cells.** (A-H) Cell viability was measured after treated with auranofin or rapamycin alone or their combination (added in a cotreatment manner) for 24 h, combination index (CI) values were calculated from the MTT assays using Calcusyn software. (I-P) Cell viability was measured after treated with auranofin or everolimus alone or their combination for 24 h, combination index (CI) values were calculated from the MTT assays using Calcusyn software. (Q-X) Cell viability was measured after treated with auranofin or AZD8055 alone or their combination for 24 h, combination index (CI) values were calculated from the MTT assays using Calcusyn software. Data from three technical replicates.

We first investigated the combined effects of mTOR and TrxR inhibitors on the viability of human gastric and colon cancer cell lines. In our study, we selected auranofin (AF) dose as it exhibits 20%-30% growth inhibition in monotherapy for combination experiment. Within 24 hours, mTOR inhibitors rapamycin, everolimus and AZD8055 inhibited cell growth as monotherapy, but the inhibitory effect was stronger when in combination with TrxR inhibitor auranofin. The combination index (CI) values were calculated from the MTT assay and revealed a synergistic growth inhibition in gastric and colon cancer cells (Figure 1A-X). Images of HCT116 cells further demonstrated the inhibitory effect of monotreatment and an increased inhibition with combination treatment (Figure S1A). By contrast, auranofin did not significantly increase the cytotoxicity of mTOR inhibitors, and the combined treatment has little effect on normal NRK-52E and MPM cells (Figure S1B-C). To confirm that the inhibiting effect was due to on-target suppression of mTOR and TrxR, we sought to evaluate additional agents. WZ26, a novel analog of curcumin, was identified as a direct TrxR inhibitor in our previous study 16. As such, WZ26 significantly killed HCT116 cells when combined with rapamycin or everolimus (Figure S1D). Piperlongumine, a structurally unrelated compound that inhibits TrxR, was also effective in this context (Figure S1E).

### mTOR and TrxR inhibitor combination increases ROS levels

**Figure 2:**
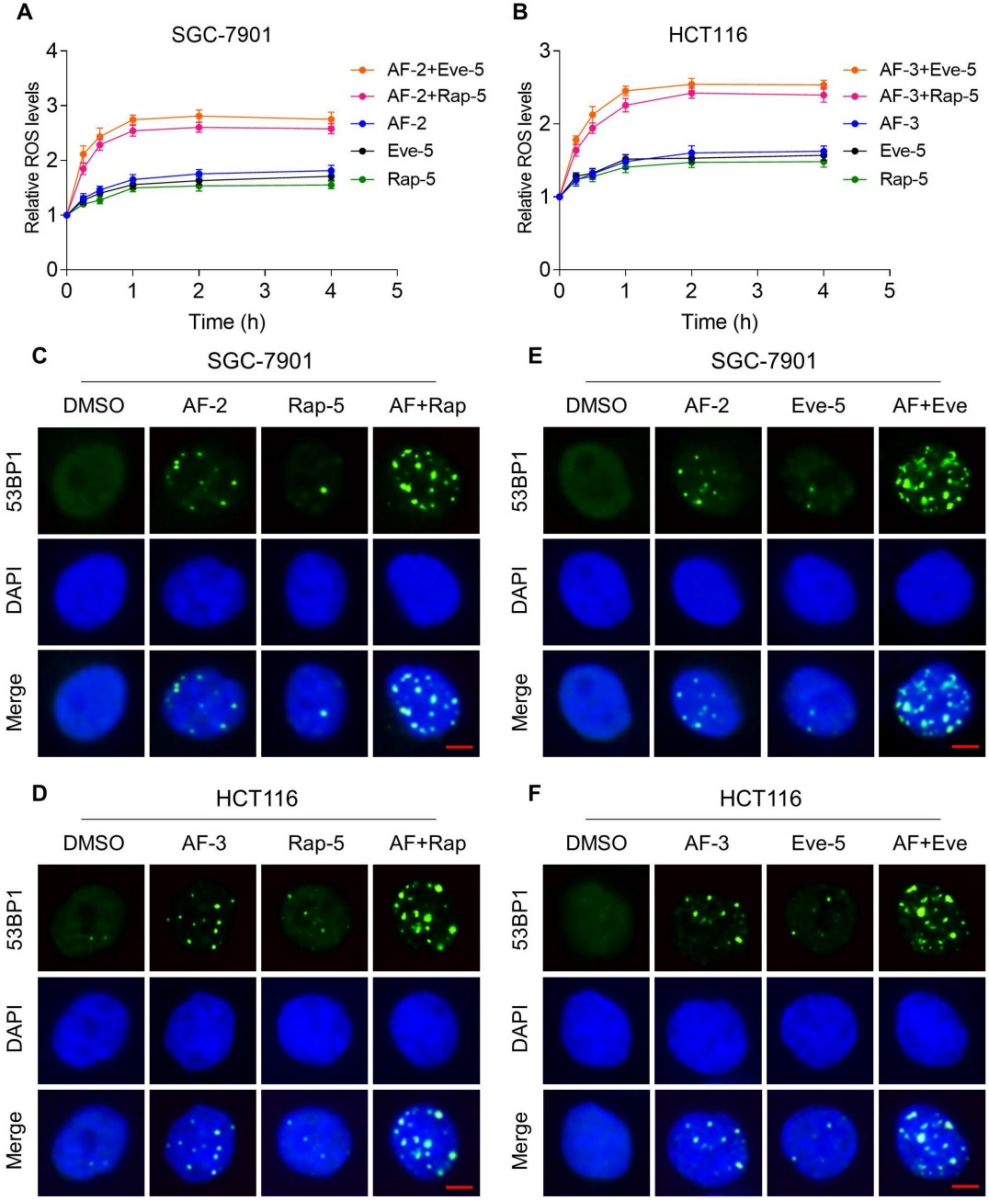
** mTOR and TrxR inhibitor combination increases ROS levels.** (A-B) Intracellular ROS levels were measured after treated with auranofin, rapamycin or everolimus alone or their combination for indicated time periods. (C) The nuclear foci formation of 53BPl was detected after treated with auranofin (2 µM) or rapamycin (5 µM) alone or their combination (2 µM auranofin and 5 µM rapamycin) for 20 h. (D) The nuclear foci formation of 53BPl was detected after treated with auranofin (3 µM) or rapamycin (5 µM) alone or their combination (3 µM auranofin and 5 µM rapamycin) for 20 h. (E-F) The nuclear foci formation of 53BPl was detected after treated with auranofin or everolimus alone or their combination for 20 h. Scale bar = 5 µm. Data from three technical replicates (* p < 0.05, ** p < 0.01).

Previous studies showed that ROS accumulation plays a critical role in the antitumor action of mTOR and TrxR inhibitors 27, 30, 31. To facilitate clinical translation, the FDA-approved drug auranofin was used for further study. We then determined whether mTOR inhibitors and auranofin were cooperatively enhancing ROS levels in gastric and colon cancer cells. Time-course results showed that mTOR inhibitors and auranofin combination markedly induced ROS generation in SGC-7901 and HCT116 cells (Figure 2A-B). Moreover, compared with rapamycin, everolimus or auranofin treatment alone, the combined treatment significantly increased ROS levels in SGC-7901 and HCT116 cells (Figure 2A-B). By contrast, the combined treatment did not cause a significant increase in ROS levels in normal NRK-52 cells (Figure S2A-B).

**Figure 3:**
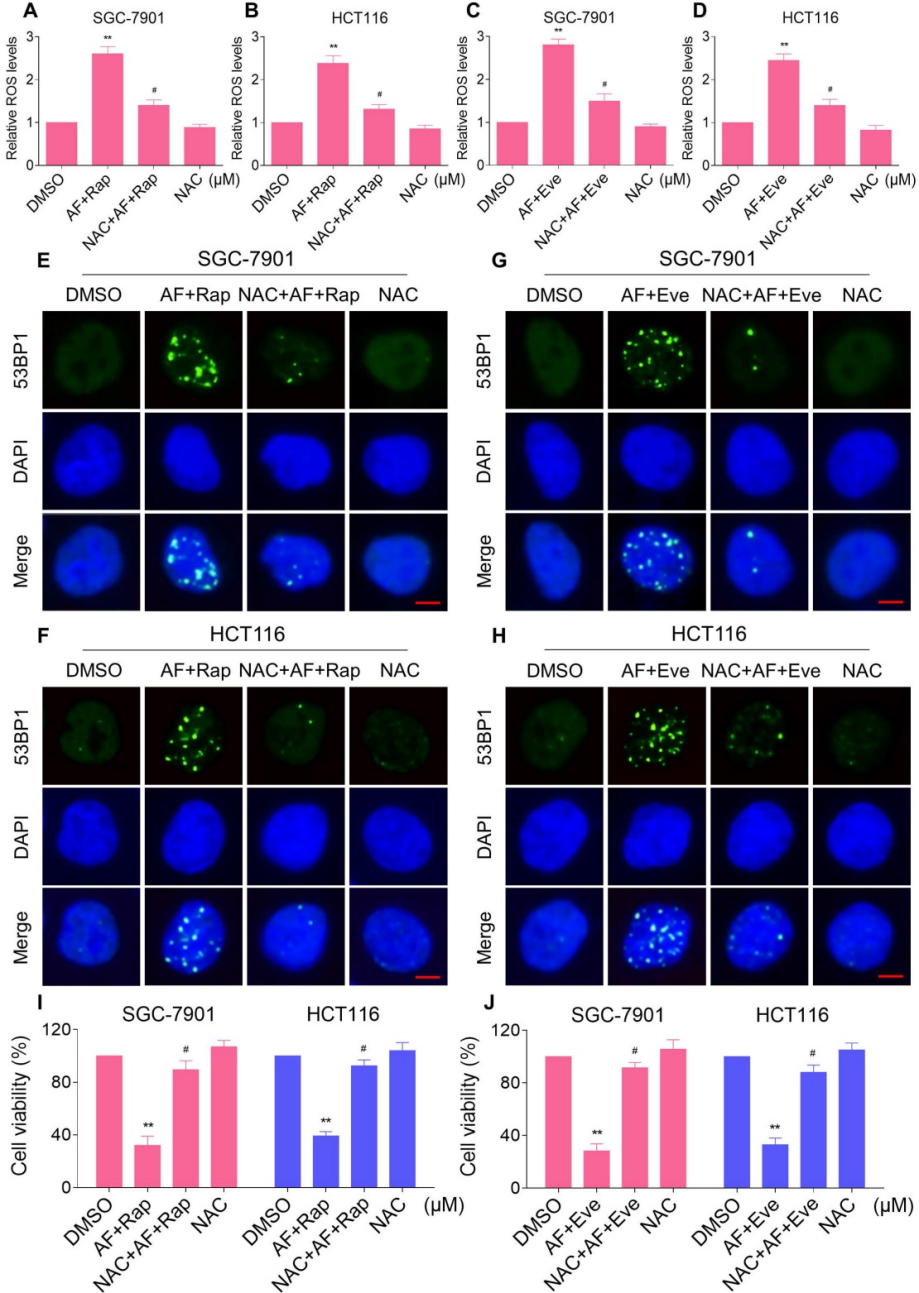
** NAC pretreatment greatly attenuated the combined treatment-induced cell death.** (A) Cells were pretreated with NAC (5 mM) for 2 h and intracellular ROS levels were measured after treated with the combination (2 µM auranofin and 5 µM rapamycin) for 2 h. (B) Cells were pretreated with NAC (5 mM) for 2 h and intracellular ROS levels were measured after treated with the combination (3 µM auranofin and 5 µM rapamycin) for 2 h. (C-D) Cells were pretreated with NAC (5 mM) for 2 h and intracellular ROS levels were measured after treated with the combination for 2 h. (E-H) Cells were pretreated with NAC (5 mM) for 2 h and nuclear foci formation of 53BPl was detected after treated with the combination for 20 h. (I-J) Cells were pretreated with NAC (5 mM) for 2 h and cell viability was measured after treated with the combination for 24 h. Scale bar = 5 µm. Data from three technical replicates (* p < 0.05, ** p < 0.01).

Excessive amounts of ROS can cause oxidative damage to lipids and DNA 32, 33. Using an immunofluorescence assay, we found that the combined treatment resulted in a significant accumulation of nuclear 53BP1 foci in SGC-7901 and HCT116 cells (Figure 2C-F). To determine the role of ROS in the combined treatment-induced cell death, we employed a widely used antioxidant, NAC, to scavenge ROS and examine the effects on these cells. Indeed, we found that pretreatment with NAC markedly reversed auranofin, everolimus and the combined treatment-induced ROS accumulation in SGC-7901 and HCT116 cells (Figure 3A-D and Figure S3A-B). Moreover, the combined treatment-induced accumulation of nuclear 53BP1 foci was markedly abolished by NAC pretreatment in both cell lines (Figure 3E-H). Importantly, NAC pretreatment greatly attenuated the combined treatment-induced cell death in both SGC-7901 and HCT116 cells (Figure 3I-J). These data suggest that ROS generation plays a critical role in the combined treatment-induced cytotoxicity in gastric and colon cancer cells.

### mTOR and TrxR inhibitor cooperate to trigger ROS-dependent ER stress

**Figure 4:**
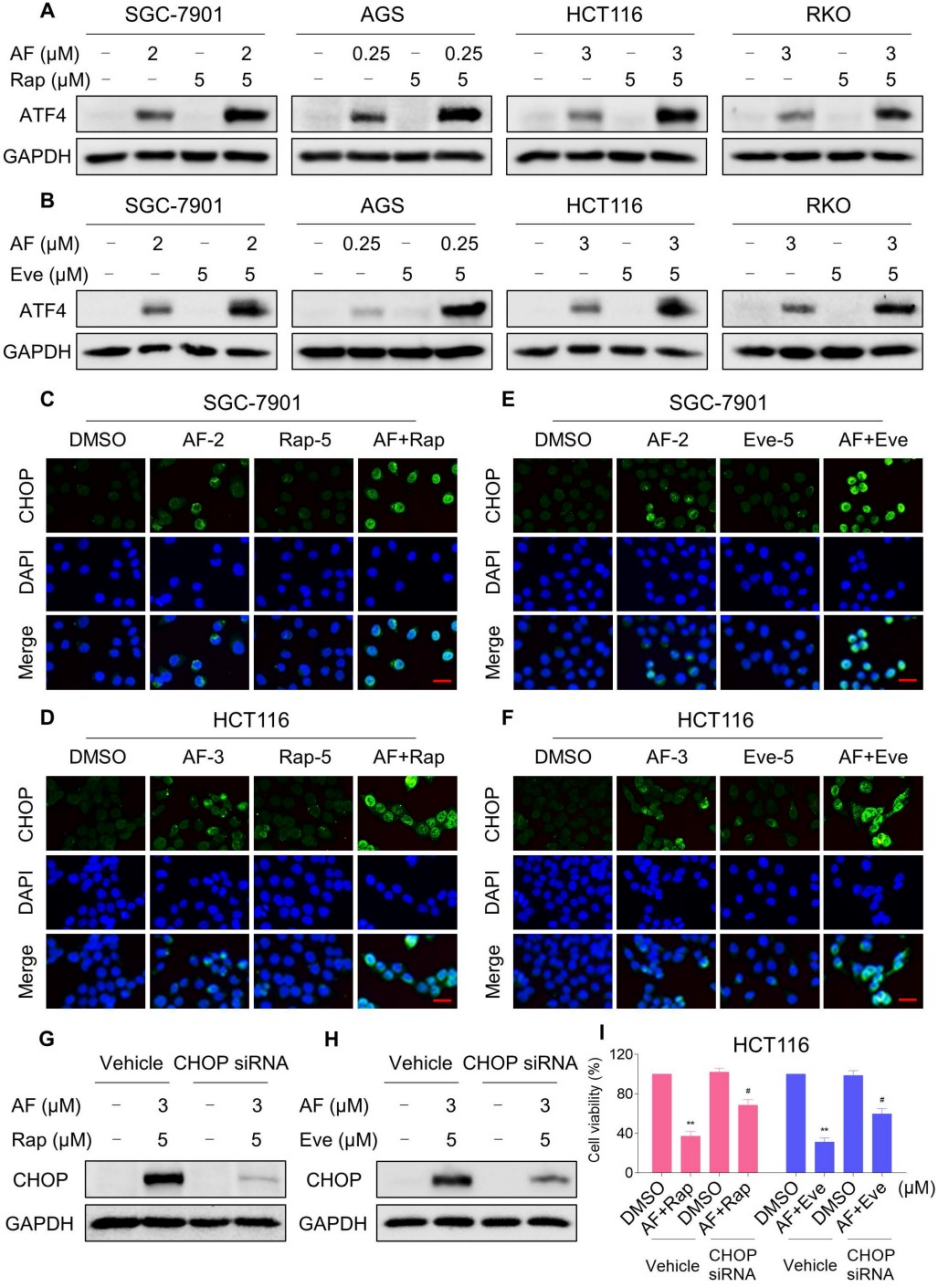
** mTOR and TrxR inhibitor cooperate to trigger ER stress.** (A) Cells were treated with auranofin or rapamycin alone or their combination for 6 h and then lysed for Western blot analyses with the indicated antibodies. (B) Cells were treated with auranofin or everolimus alone or their combination for 6 h and then lysed for Western blot analyses with the indicated antibodies. (C) Cells after treatment with auranofin (2 µM) or rapamycin (5 µM) alone or their combination (2 µM auranofin and 5 µM rapamycin) for 12 h were stained with CHOP and were then analyzed by fluorescence microscope. (D) Cells after treatment with auranofin (3 µM) or rapamycin (5 µM) alone or their combination (3 µM auranofin and 5 µM rapamycin) for 12 h were stained with CHOP and were then analyzed by fluorescence microscope. (E-F) Cells after treatment with auranofin or everolimus alone or their combination for 12 h were stained with CHOP and were then analyzed by fluorescence microscope. (G-H) HCT116 cells were infected with CHOP siRNA, CHOP expression in HCT116 cells was determined by Western blot analyses after stimulation with the combination for 12 h. (I) HCT116 cells transfected with CHOP siRNA were treated with the combination for 24 h. Cell viability was measured using a methyl thiazolyl tetrazolium assay. Scale bar = 25 µm. Data from three technical replicates (* p < 0.05, ** p < 0.01).

**Figure 5:**
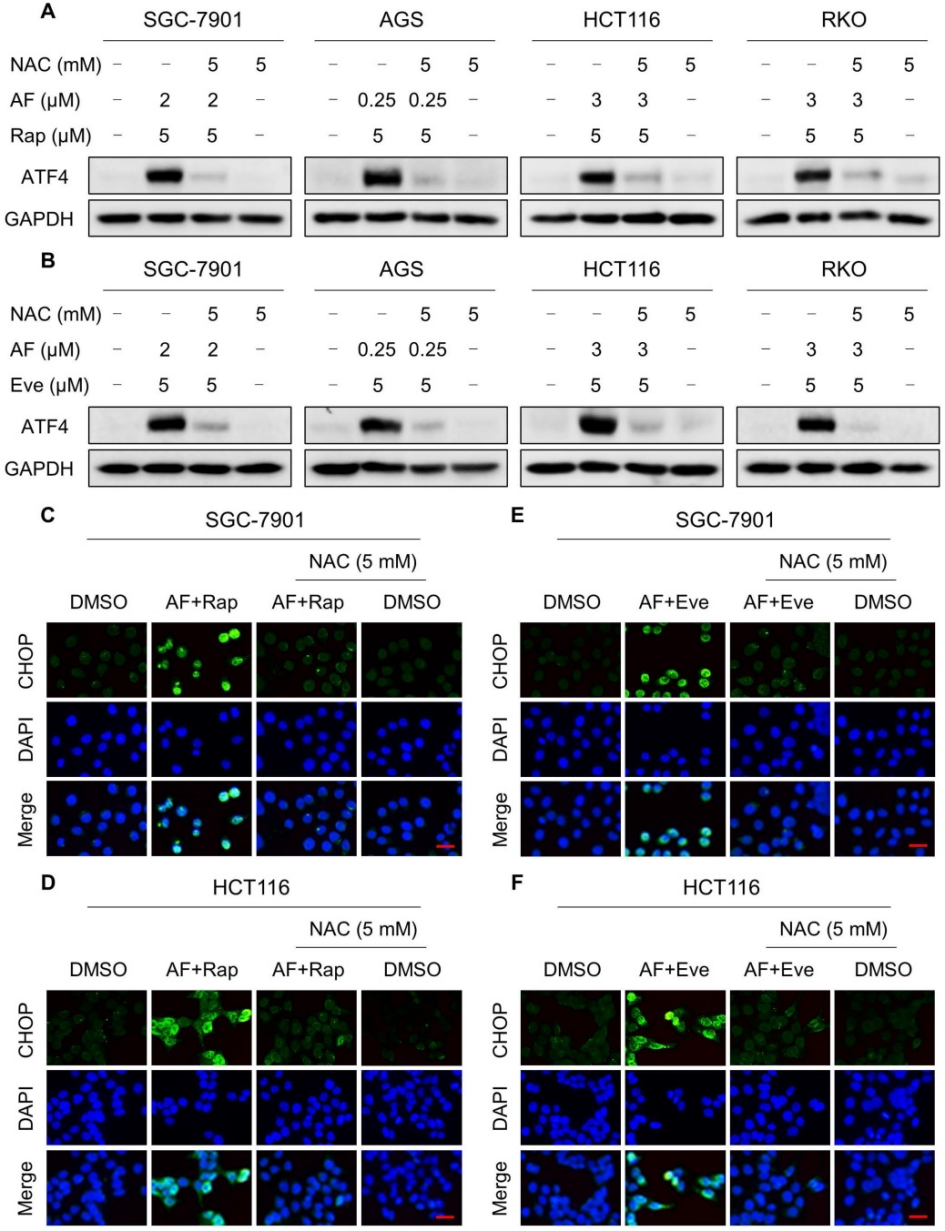
** NAC pretreatment significantly reversed the combined treatment-induced ER stress.** (A-B) Cells were pretreated with NAC (5 mM) for 2 h and cell lysates were blotted with the indicated antibodies after treated with the combination for 6 h. (C-D) Cells were pretreated with NAC (5 mM) for 2 h and CHOP expression was observed by fluorescence microscope after treated with auranofin and rapamycin combination for 12 h. (E-F) Cells were pretreated with NAC (5 mM) for 2 h and CHOP expression was observed by fluorescence microscope after treated with auranofin and everolimus combination for 12 h. Scale bar = 25 µm. Data from three technical replicates.

Our previous study showed that the accumulation of ROS could trigger cell death via activating ER stress pathway 17. To investigate the underlying mechanisms of the combinatorial effect of mTOR and TrxR inhibitor, we analyzed the expression of ER stress-related proteins in cancer cells after treated with mTOR inhibitors and auranofin. Activating transcription factor 4 (ATF4) and phosphorylation of eukaryotic initiation factor-2α (eIF2α) are excellent readouts of ER stress pathway activation. Our results showed that treatment with auranofin alone increased ATF4 and p-eIF2α expression, which was consistent with our previous report (Figure 4A-B and Figure S4A-B). Interestingly, combination of auranofin and mTOR inhibitors triggered a significantly greater increase in ATF4 and p-eIF2α expressions (Figure 4A-B and Figure S4A-B). CHOP has been considered as a central mediator of ER stress-induced cell death 34. Using an immunofluorescence assay, we found that the combined treatment significantly increased CHOP expression in the nucleus. Although auranofin treatment effectively increased CHOP expression compared to the mTOR inhibitor, combination treatment is more effective on CHOP increasement than auranofin treatment alone (Figure 4C-F). To determine whether CHOP was crucial for the therapeutic effects of mTOR inhibitors and auranofin combination, the CHOP gene was silenced using siRNA in HCT116 cells. After transfected with CHOP siRNA, CHOP expression induced by the combination of mTOR inhibitors and auranofin was markedly abolished (Figure 4G-H), but it did not affect the expression of ATF4 (Figure S4C-D), demonstrating that the siRNA is specific for CHOP. Most importantly, the cooperative effect of mTOR inhibitors and auranofin on cell death was significantly reversed, suggesting that CHOP upregulation is essential for the therapeutic effects of this combination (Figure 4I). Notably, the elevated levels of ATF4 and CHOP were significantly abolished after NAC pretreatment (Figure 5A-F). These results provided further support that antitumor efficacy of mTOR inhibitors and auranofin combination is dependent on ROS accumulation.

### Elevated JNK signaling contributes to the therapeutic effects of mTOR and TrxR inhibitor

**Figure 6:**
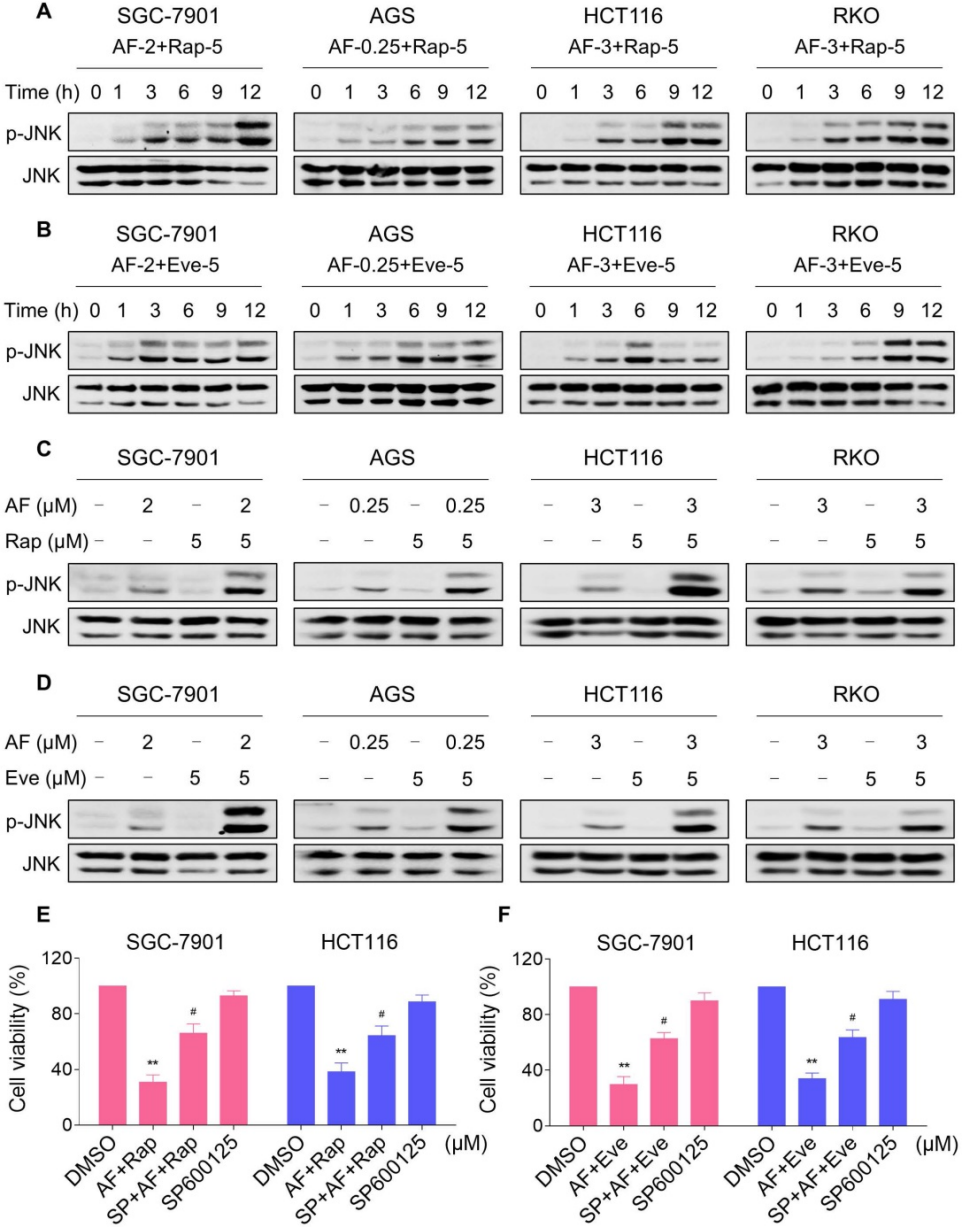
**mTOR and TrxR inhibitor cooperate to activate JNK signaling pathway.** (A-B) Cells were treated with the combination for indicated time periods and then lysed for Western blot analyses with the indicated antibodies. (C) Cells were treated with auranofin or rapamycin alone or their combination for 12 h and then lysed for Western blot analyses with the indicated antibodies. (D) SGC-7901, AGS and RKO cells were treated with auranofin or everolimus alone or their combination for 12 h and then lysed for Western blot analyses with the indicated antibodies. HCT116 cells were treated with auranofin or everolimus alone or their combination for 6 h (based on the time-course result) and then lysed for Western blot analyses with the indicated antibodies. (E-F) Cells were pretreated with SP600125 (20 µM) for 2 h and cell viability was measured after treated with the combination for 24 h. Data from three technical replicates (* p < 0.05, ** p < 0.01).

**Figure 7:**
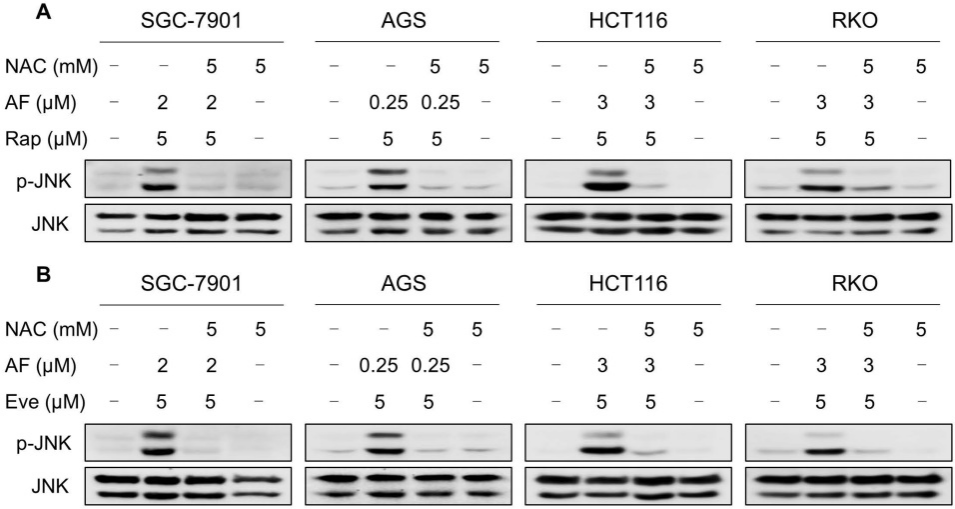
** NAC pretreatment significantly reversed the combined treatment-induced activation of JNK signaling pathway.** (A) Cells were pretreated with NAC (5 mM) for 2 h and cell lysates were blotted with the indicated antibodies after treated with auranofin and rapamycin combination for 12 h. (B) SGC-7901, AGS and RKO cells were pretreated with NAC (5 mM) for 2 h and cell lysates were blotted with the indicated antibodies after treated with auranofin and everolimus combination for 12 h. HCT116 cells were pretreated with NAC (5 mM) for 2 h and cell lysates were blotted with the indicated antibodies after treated with auranofin and everolimus combination for 6 h. Data from three technical replicates.

JNK signaling can be activated in response to a variety of stimuli, including cytokines, irradiation and oxidative stress 35-37. Therefore, we set out to determine whether the JNK signaling pathway was activated in cancer cells after treated with mTOR inhibitors and auranofin. Time-course results showed that mTOR inhibitors and auranofin combination significantly increased the phosphorylation of JNK in SGC-7901, AGS, HCT116 and RKO cells (Figure 6A-B). Moreover, compared with rapamycin, everolimus or auranofin treatment alone, the combined treatment caused a significantly greater increase in the phosphorylation of JNK in these cells (Figure 6C-D). To determine whether JNK signaling pathway was crucial for the combined treatment-induced cell death, the phosphorylation of JNK was inhibited using the specific JNK inhibitor SP600125. Indeed, the phosphorylation of JNK induced by the combination of mTOR inhibitors and auranofin was greatly reversed by SP600125 pretreatment (Figure S5A-B). This was associated with an appreciable reduction in the combined treatment-induced cell death in SGC-7901 and HCT116 cells, indicating that JNK activation is essential for the therapeutic effects of this combination (Figure 6E-F). Notably, the elevated phosphorylation of JNK was significantly abolished after NAC pretreatment, indicating that activation of the JNK signaling pathway is due to accumulation of intracellular ROS in these cells (Figure 7A-B).

### mTOR and TrxR inhibitor cooperate to induce autophagy

**Figure 8:**
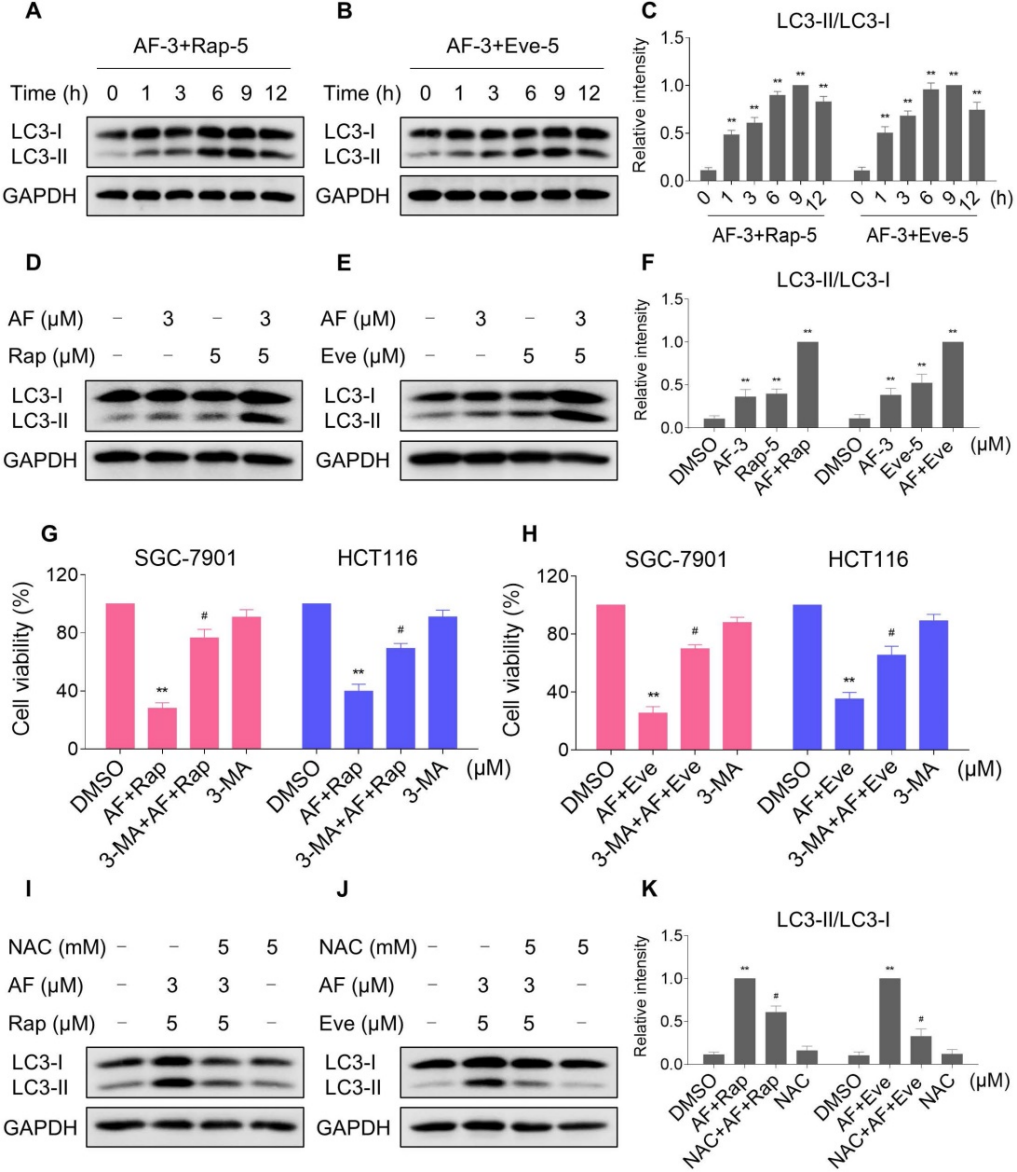
** mTOR and TrxR inhibitor cooperate to induce autophagy.** (A-C) Cells were treated with the combination for indicated time periods and then lysed for Western blot analyses with the indicated antibodies. (D and F) Cells were treated with auranofin or rapamycin alone or their combination for 9 h and then lysed for Western blot analyses with the indicated antibodies. (E-F) Cells were treated with auranofin or everolimus alone or their combination for 9 h and then lysed for Western blot analyses with the indicated antibodies. (G-H) Cells were pretreated with 3-MA (5 mM) for 2 h and cell viability was measured after treated with the combination for 24 h. (I-K) Cells were pretreated with NAC (5 mM) for 2 h and cell lysates were blotted with the indicated antibodies after treated with the combination for 9 h. Data from three technical replicates (* p < 0.05, ** p < 0.01).

Autophagy is a catabolic process in which autophagosomes is formed, and it has double-sided effects on cell proliferation and death. Recent studies have reported that excessive production of ROS can induce autophagy 38, 39. During autophagy, LC3 is converted from LC3-I to LC3-II and is positioned on the membrane of autophagosomes 40. We then analyzed the levels of LC3-I/II in HCT116 cells after treated with mTOR inhibitors and auranofin. Time-course results showed that mTOR inhibitors and auranofin combination significantly increased the ratio of LC3-II to LC3-I (Figure 8A-C). Moreover, compared with rapamycin, everolimus or auranofin treatment alone, the combined treatment caused a significantly greater increase in the ratio of LC3-II to LC3-I (Figure 8D-F). We further transfected the tandem monomeric RFP-GFP-tagged LC3 lentivirus into HCT116 cells to determine the effect of everolimus and auranofin on autophagic flux. Combination of everolimus and auranofin significantly increased the colocalization of GFP-LC3 and RFP-LC3 puncta in HCT116 cells, indicating increased autophagic flux in HCT116 cells (Figure S6A). To determine whether autophagy was responsible for the combined treatment-induced cell death, the cells were treated with the combination of mTOR inhibitors and auranofin after pretreated with 3-methyladenine (3-MA), an autophagy inhibitor. Indeed, 3-MA pretreatment significantly attenuated the combined treatment-induced cell death in both SGC-7901 and HCT116 cells, suggesting that autophagy activation is essential for the therapeutic effects of this combination (Figure 8G-H). Notably, the increase in the ratio of LC3-II to LC3-I was significantly abolished after NAC pretreatment, indicating that activation of autophagy in both cells is due to accumulation of intracellular ROS (Figure 8I-K).

**Figure 9:**
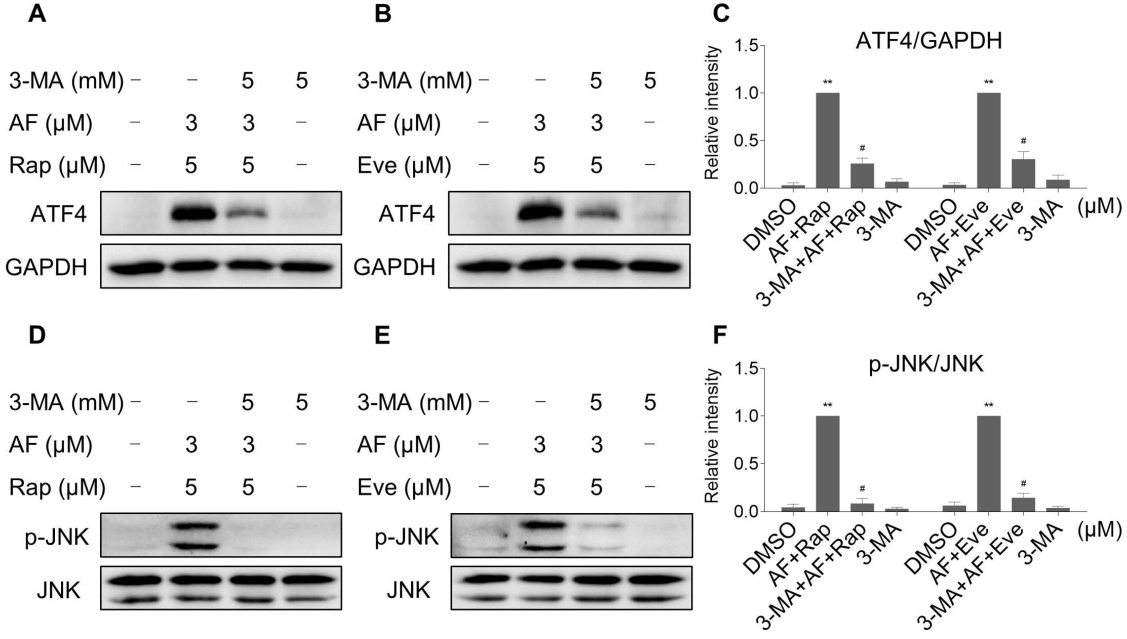
** 3-MA pretreatment significantly reversed the combined treatment-induced expression of ATF4 and p-JNK.** (A and C) HCT116 cells were pretreated with 3-MA (5 mM) for 2 h and cell lysates were blotted with the indicated antibodies after treated with auranofin and rapamycin combination for 6 h. (B-C) HCT116 cells were pretreated with 3-MA (5 mM) for 2 h and cell lysates were blotted with the indicated antibodies after treated with auranofin and everolimus combination for 6 h. (D and F) HCT116 cells were pretreated with 3-MA (5 mM) for 2 h and cell lysates were blotted with the indicated antibodies after treated with auranofin and rapamycin combination for 12 h. (E-F) HCT116 cells were pretreated with 3-MA (5 mM) for 2 h and cell lysates were blotted with the indicated antibodies after treated with auranofin and everolimus combination for 6 h. Data from three technical replicates (* p < 0.05, ** p < 0.01).

Interestingly, 3-MA was more effective in reversing the cooperative effect of mTOR inhibitors and auranofin on cell death compared with JNK inhibitor SP600125 or CHOP siRNA. Therefore, we hypothesized that autophagy may act as an upstream event involved in the combined treatment-induced activation of ER stress and JNK signaling pathway. Indeed, we observed that 3-MA pretreatment significantly attenuated the combined treatment-induced expression of ATF4 (Figure 9A-C). Moreover, the phosphorylation of JNK induced by the combination of mTOR inhibitors and auranofin was greatly reversed by 3-MA pretreatment (Figure 9D-F).

### TrxR expression and activity are up-regulated by mTOR inhibitors

**Figure 10:**
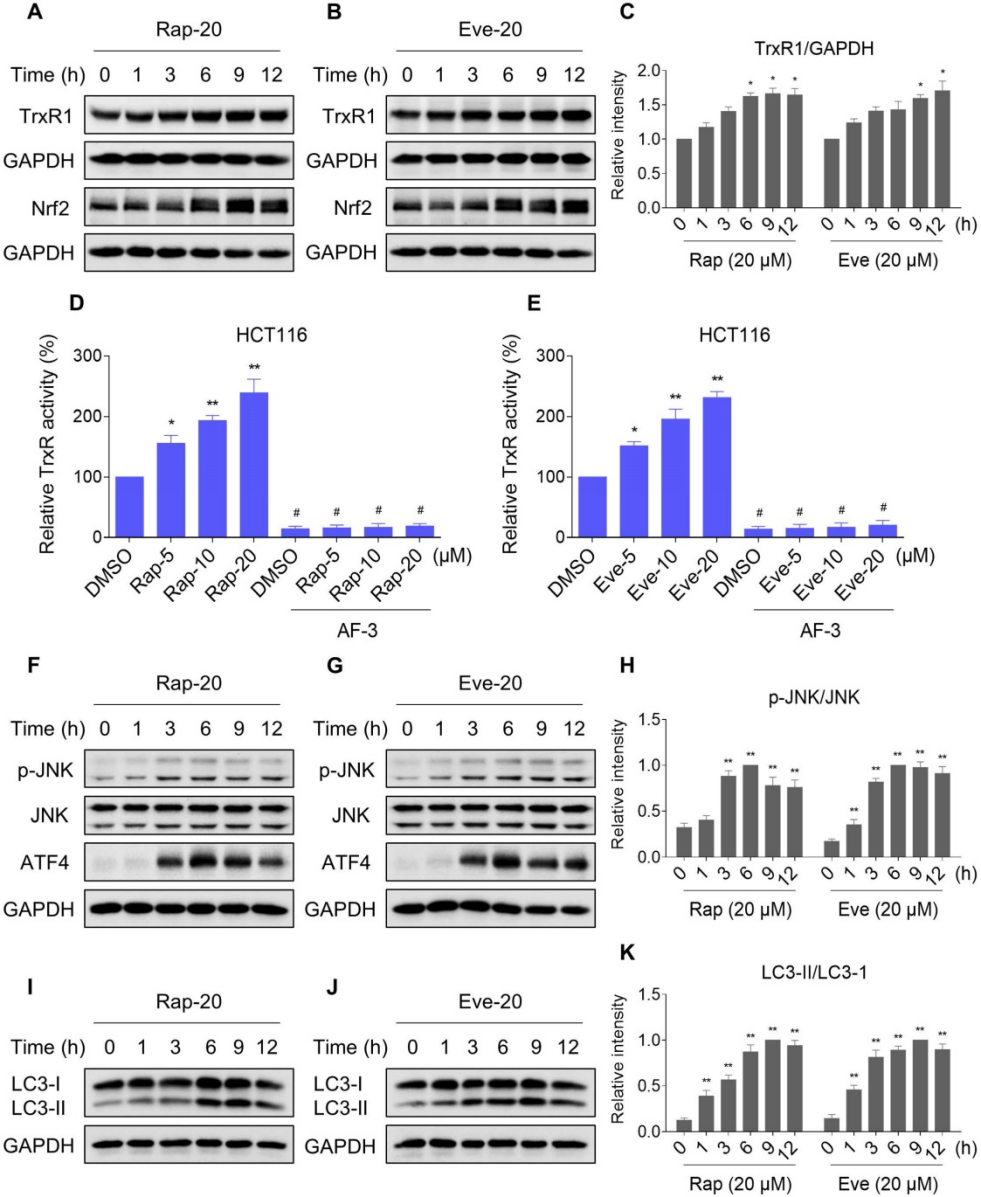
** TrxR expression and activity are up-regulated by mTOR inhibitors.** (A-C) HCT116 cells were treated with rapamycin (20 µM) or everolimus (20 µM) for indicated time periods and then lysed for Western blot analyses with the indicated antibodies. (D) TrxR activity was measured by the endpoint insulin reduction assay after treated with rapamycin or auranofin alone or their combination. (E) TrxR activity was measured by the endpoint insulin reduction assay after treated with everolimus or auranofin alone or their combination. (F-K) HCT116 cells were treated with rapamycin (20 µM) or everolimus (20 µM) for indicated time periods and then lysed for Western blot analyses with the indicated antibodies. Data from three technical replicates (* p < 0.05, ** p < 0.01).

Our results showed that rapamycin and everolimus can induce ROS generation at a concentration of 5 μM, but have little effect on cell death and activation of ER stress and JNK signaling pathway. To further investigate the mechanistic basis of mTOR inhibitors and auranofin combination therapy, we sought to examine the expression of ROS-related proteins in HCT116 cells after treated with mTOR inhibitors. Notably, the expression of antioxidant proteins TrxR1 and Nrf2 were increased in response to rapamycin or everolimus monotherapy (Figure 10A-C). Immunofluorescence assay showed that the expression of Nrf2 and nuclear Nrf2 was significantly increased in response to everolimus (Figure S7A). Moreover, the activity of TrxR was also increased after treatment with rapamycin or everolimus, and the increase of TrxR activity was reversed by the addition of auranofin (Figure 10D-E). These findings indicate that TrxR induction is an adaptive response to mTOR inhibitors. To expand our observations to the stimulation of mTOR inhibitors, we also analyzed the expression of ATF4, p-JNK and LC3-I/II in HCT116 cells after treated with mTOR inhibitors. Time-course results showed that rapamycin and everolimus significantly increased the expression of ATF4 and p-JNK (Figure 10F-H). Moreover, the ratio of LC3-II to LC3-I was greatly increased in response to rapamycin or everolimus alone (Figure 10I-K). These results further support the notion that activation of ER stress, JNK signaling and autophagy is essential for the therapeutic effects of mTOR inhibitors and auranofin combination.

### mTOR and TrxR inhibitor synergize to inhibit tumor growth in nude mice

**Figure 11:**
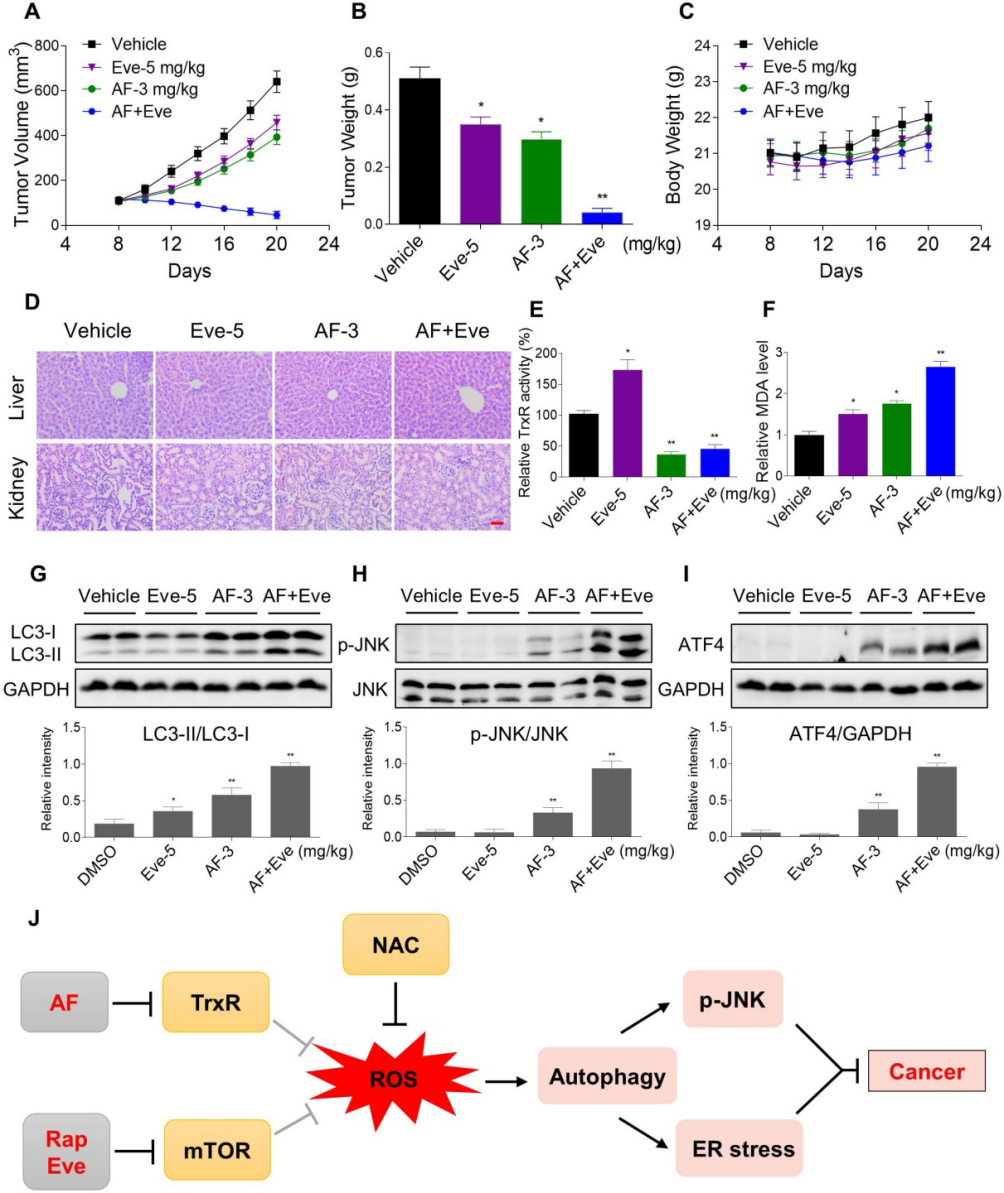
** Everolimus and auranofin cooperate to inhibit tumor growth in nude mice.** (A) Everolimus (5 mg/kg) and auranofin (3 mg/kg) combined treatment significantly decreased tumor volume and tumor weight (B) of HCT116 human colon cancer xenografts in nude mice, but did not significantly affect body weight (C) of mice. (D) HE staining of the major organs. (E) The TrxR activity in tumor tissues. (F) MDA levels in tumor tissues. (G-I) The protein levels of LC3-I, LC3-II, p-JNK, JNK, ATF4 and GAPDH were determined by Western blot analyses. (J) The proposed working model. Scale bar = 75 µm (* p < 0.05, ** p < 0.01).

To extend these *in vitro* findings to *in vivo* tumor growth, we evaluated the combination of everolimus and auranofin in a subcutaneous xenograft model of HCT116 cells in immunodeficient mice. The mice were equally divided into four groups (six mice/group) and received the following treatments: (1) control vehicle; (2) everolimus (5 mg/kg); (3) auranofin (3 mg/kg); (4) everolimus (5 mg/kg) plus auranofin (3 mg/kg). After 13 days of treatment, we found that 5 mg/kg everolimus or 3 mg/kg auranofin inhibited the growth of HCT116 xenografts (Figure 11A-B). Remarkably, the combined treatment with everolimus and auranofin showed stronger inhibitory effect on the growth of HCT116 xenografts with no significant signs of toxicity (Figure 11A-D). Similar results were also observed from subcutaneous xenograft model of SGC-7901 cells in immunodeficient mice (Figure S8A-C). Of note, we noticed that tumor growth inhibition was durable even after stopping the combination treatment (Figure S8A). Mechanistically, we found that the TrxR activity was increased in the everolimus group, but significantly reduced in the combination group (Figure 11E). In addition, everolimus in combination with auranofin markedly increased the level of malondialdehyde (MDA), a marker of oxidative stress, in the tumor tissues (Figure 11F). Moreover, the combined treatment significantly increased the ratio of LC3-II to LC3-I, and the expression levels of ATF4 and p-JNK (Figure 11G-I). These *in vivo* data support our findings in cell culture experiments and further strengthen the hypotheses that the generation of ROS is critical for the synergistic effect of everolimus and auranofin (Figure 11J).

## Discussion

The progress toward inhibiting specific oncogenic drivers in cancer has been hampered by both intrinsic and acquired resistance. The mTOR pathway is dysregulated in several human disorders, including cancer 22. Owing to the pervasiveness of mTOR activation in human cancers, there has been a continuing interest in mTOR inhibitors for cancer therapy 21, 41. Several mTOR inhibitors have already undergone clinical trials for treating cancers. However, mTOR-targeted cancer therapies still face great challenges due to the anticancer efficacies of mTOR inhibitor monotherapy are limited in clinical treatment 24, 42-44. These findings indicate that more effective therapeutic strategies are required for the treatment of cancers. One strategy may be to simultaneously inhibit key oncogenic pathways and other cancer-specific vulnerabilities. Here, we demonstrate that mTOR inhibitors and auranofin synergize to induce cancer cell death in gastric and colon cancer cells, whilst has little effect on normal NRK-52E and MPM cells, suggesting that this combination is not generally toxic. Importantly, we show that combination treatment with auranofin and everolimus significantly suppresses tumor growth in HCT116 and SGC-7901 xenograft models with no significant signs of toxicity.

Under physiological conditions, ROS production and elimination is tightly regulated. Compared with normal cells, cancer cells usually generate and maintain higher ROS levels due to distorted metabolism 5, 45. Elevated ROS levels render cancer cells more sensitive to agents that increase ROS generation. Therefore, manipulating ROS levels by redox modulation is an effective strategy to selectively kill cancer cells 4, 46. In this study, we show that combination of auranofin and mTOR inhibitors resulted in a significant increase in intracellular ROS levels, and that pretreatment with NAC significantly reversed the combined-induced ROS generation and cell death, indicating that ROS play a critical role in the antitumor activity of this combination. Importantly, the combined treatment did not cause a significant increase in intracellular ROS levels in normal NRK-52 cells. These data further support the notion that stimulation of ROS is a potential therapeutic strategy for cancer treatment.

Although inhibition of mTOR pathway is being pursued to impair the viability of cancer cells, but current inhibitors of mTOR are limited by issues of potency and resistance 23. Our findings show that the expression and activity of TrxR in HCT116 cells were increased after treatment with rapamycin or everolimus alone. Moreover, we found that auranofin could block the increase of TrxR activity caused by mTOR inhibitors, and significantly increases the cytotoxicity of mTOR inhibitors in cancer cells. These data suggest that TrxR induction might be an adaptive response to mTOR inhibitors, and TrxR inhibition is an effective pharmacologic approach for increasing therapeutic efficacy of mTOR inhibitors to cancer cells. Previous studies have reported that auranofin could interact with TrxR and inhibits its activity 47, 48. In addition, recent studies have shown that various molecules, signal transduction pathways and mitochondrial function-related proteins, including NONO, p53 and PI3K/AKT/mTOR signaling pathway are involved in the process of auranofin-induced cell death 49-51. Therefore, further study is needed to establish whether TrxR induction is a resistance mechanism to mTOR inhibitors. Extending these observations, we additionally observed increased expression of antioxidant transcription factor Nrf2 in HCT116 cells after treated with rapamycin or everolimus alone for 6 h. In addition, the nuclear translocation of Nrf2 was also increased in response to everolimus, indicating Nrf2 might also be an adaptive response to mTOR inhibitors. Although the majority of this study has focused on TrxR, the role of Nrf2 is warranted for further investigation.

Autophagy has been shown to promote either cell survival or death depending on cell type and stimulus 52-54. In the present study, we found that mTOR inhibitors or auranofin treatment alone induced autophagy in colon cancer cells, while the inductive effect was stronger when used in combination. We further demonstrate that increased autophagy upon mTOR inhibitors and auranofin combination treatment promoted cancer cell death rather than cell survival. Notably, the increased autophagy induced by the combination of mTOR inhibitors and auranofin was significantly abolished after NAC pretreatment, indicating that activation of autophagy is due to accumulation of intracellular ROS in colon cancer cells, but the precise mechanism underlying this relationship remains unclear. Further studies will be required to fully understand the relationship between ROS generation and autophagy in the combined treatment-induced cancer cell death.

Another interesting finding from our study is that both ER stress and JNK signaling pathways are significantly activated in response to mTOR inhibitors and auranofin combination treatment, and these activations are essential for the therapeutic effects of combination treatment. Recent studies found that ROS accumulation is an essential component of the event leading to ER stress-mediated cell death 55, 56. ROS has also been reported to activate JNK pathway leading us to speculate that ROS may be the common upstream mediator of ER stress and JNK signaling pathway 57. Indeed, we demonstrated that combination treatment induced activation of ER stress and JNK is significantly abolished after NAC pretreatment. These data strongly suggest that ROS acts upstream of ER stress and JNK signaling pathway. In addition, autophagy inhibitor attenuated the combined treatment-induced expression of ATF4 and p-JNK, suggesting that autophagy may act as an upstream event involved in the combined treatment-induced activation of ER stress and JNK signaling pathways.

## Conclusions

Currently, there are no effective treatments for gastric and colon cancers. Herein we demonstrate that that mTOR inhibitors and auranofin synergize to induce cancer cell death by inducing oxidative stress. Fortunately, rapamycin, everolimus and auranofin are currently being evaluated in numerous clinical trials to treat cancer (clinicaltrials.gov). Therefore, tolerable doses of relevant drug combinations will be available in the near future. Our study provided a promising therapeutic combination for cancer treatment and revealed that selectively enhancing ROS generation might be an effective strategy to eliminate cancer cells.

## Supplementary Material

Supplementary figures and tables.

## Abbreviations

AF: Auranofin
ROS: reactive oxygen species
mTOR: mammalian target of rapamycin
TrxR: thioredoxin reductase
JNK: c-Jun N-terminal Kinase
NAC: N-acetyl-L-cysteine
DCFH-DA: 2′,7′-dichlorofluorescin diacetate
MDA: malondialdehyde
CI: combination index
