# Supplementary material for: Compensatory combination of mTOR and TrxR inhibitors to cause oxidative stress and regression of tumors
Source: Theranostics. 2021 Feb 25;11(9):4335–50. doi: 10.7150/thno.52077 (PMC7977446; doi:10.7150/thno.52077)
Supplement: Supplementary file 1 — Supplementary figures and tables. [file thnov11p4335s1.pdf]

Supporting Information

Compensatory combination of mTOR and TrxR inhibitors to cause oxidative stress and regression of tumors

Yiqun Xia[1#], Jundixia Chen[2#], Yun Yu[2#], Fengjiao Wu[2#], Xin Shen[2], Chenyu Qiu[2], Tingting Zhang[2], Lin Hong[2], Peisen Zheng[2], Rongrong Shao[2], Chenxin Xu[2], Fang Wu[1], Wei Chen[1], Congying Xie[1], Ri Cui[1,2,3,4]*, Peng Zou[1,2,4]*

[1] The First Affiliated Hospital of Wenzhou Medical University, Wenzhou Medical University, Wenzhou 325035, China

[2] Cancer and Anticancer Drug Research Center, School of Pharmaceutical Sciences, Wenzhou Medical University, Wenzhou 325035, China

[3] Biomedical Collaborative Innovation Center of Zhejiang Province, Wenzhou University, Wenzhou 325035, China

[4] Wenzhou University-Wenzhou Medical University Collaborative Innovation Center of Biomedical, Wenzhou 325035, China

* **Corresponding author:** Peng Zou, Ph.D; Address: School of Pharmaceutical Sciences, Wenzhou Medical University, Wenzhou 325035, China; Tel: +86-577-86699892; Fax: +86-577-86689982
E-mail: zoupeng@wmu.edu.cn

* Co-corresponding author: Ri Cui, Ph.D; Address: School of Pharmaceutical Sciences, Wenzhou Medical University, Wenzhou 325035, China; Tel: +86-577-86699892; Fax: +86-577-86689982
E-mail: wzmucuiri@163.com

# These authors contributed equally to this work

Supplementary Figures and Legends

Figure S1

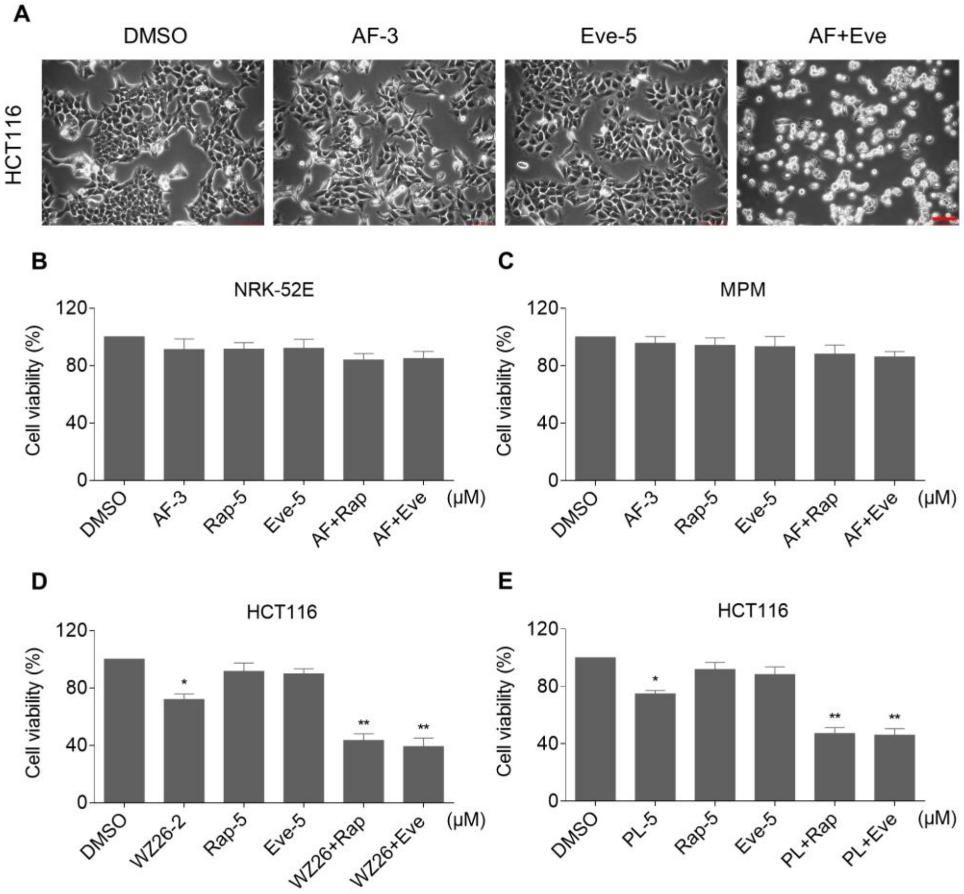

Figure S1. mTOR and TrxR inhibitors synergize to induce cell death in gastric and colon cancer cells. (A) Cell morphology was observed after treated with auranofin (3 μM) or everolimus (5 μM) alone or their combination (3 μM auranofin and 5 μM everolimus) at the indicated doses for 24 h. Scale bar = 75 μm. (B-C) Cell viability was measured after treated with auranofin, rapamycin, or everolimus alone or their combination for 24 h. (D) Cell viability was measured after treated with WZ26, rapamycin, or everolimus alone or their combination for 24 h. (E) Cell viability was measured after treated with piperlongumine (PL), rapamycin, or everolimus alone or their combination for 24 h. Data from three technical replicates. (* $p < 0.05$, ** $p < 0.01$).

Figure S2

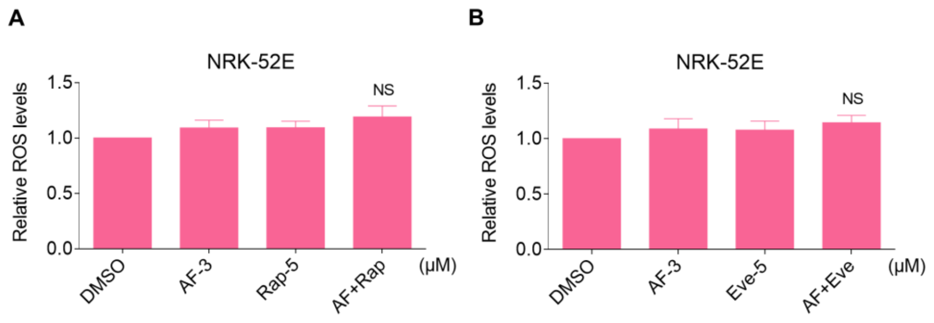

Figure S2. mTOR and TrxR inhibitor combination did not significantly increase ROS levels in NRK-52E cells. (A) Intracellular ROS levels were measured after treated with auranofin (3 μM) or rapamycin (5 μM) alone or their combination (3 μM auranofin and 5 μM rapamycin) for 2 h. (B) Intracellular ROS levels were measured after treated with auranofin (3 μM) or everolimus (5 μM) alone or their combination (3 μM auranofin and 5 μM everolimus) for 2 h. Data from three technical replicates.

Figure S3

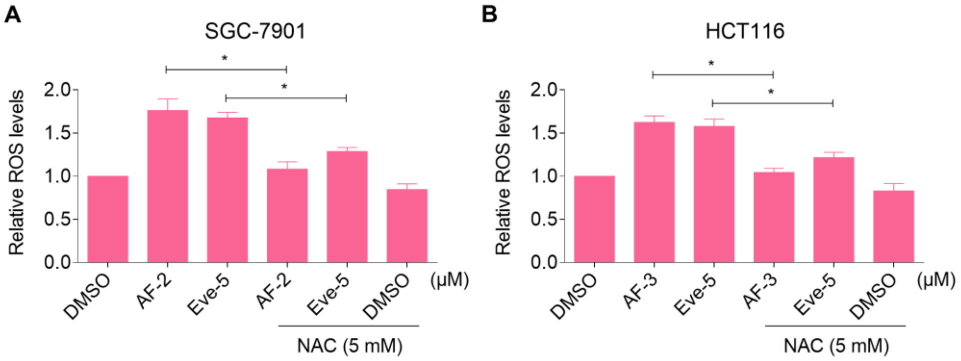

Figure S3. NAC pretreatment significantly reversed the ROS accumulation induced by auranofin or everolimus. (A) Cells were pretreated with NAC (5 mM) for 2 h and intracellular ROS levels were measured after treated with auranofin (2 μM) or everolimus (5 μM) for 2 h. (B) Cells were pretreated with NAC (5 mM) for 2 h and intracellular ROS levels were measured after treated with auranofin (3 μM) or everolimus (5 μM) for 2 h. Data from three technical replicates. (* $p < 0.05$).

Figure S4

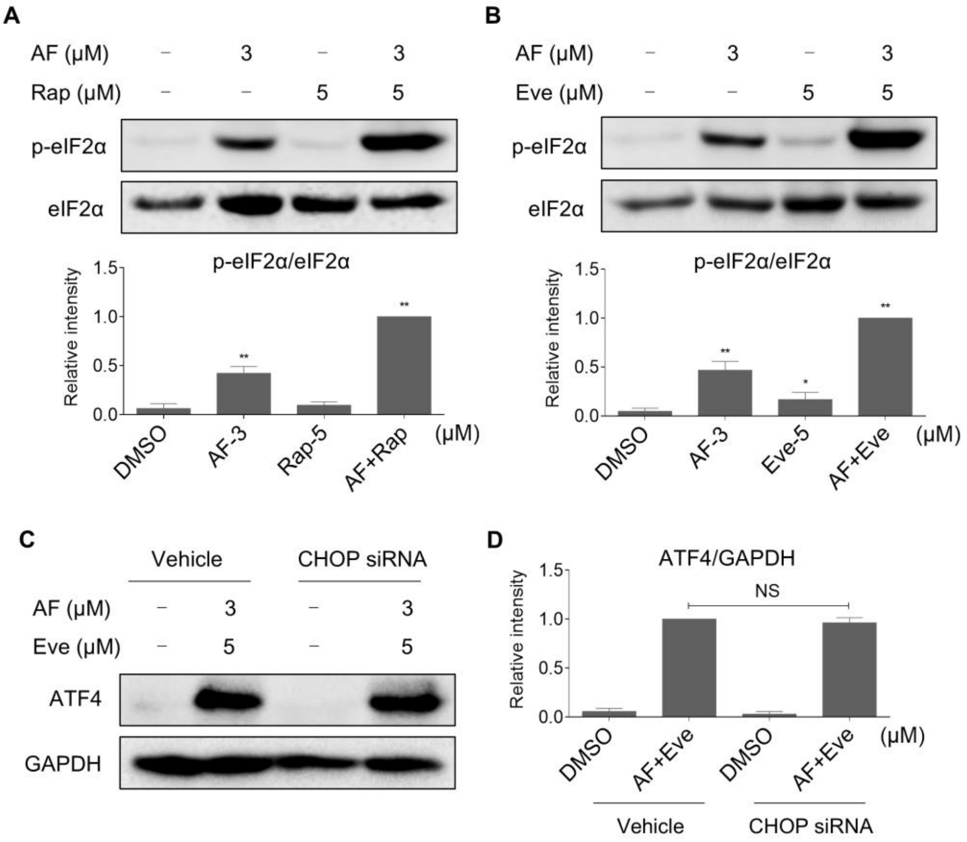

Figure S4. mTOR and TrxR inhibitor cooperate to trigger ER stress. (A) HCT116 cells were treated with auranofin or rapamycin alone or their combination for 6 h and then lysed for Western blot analyses with the indicated antibodies. (B) HCT116 cells were treated with auranofin or everolimus alone or their combination for 6 h and then lysed for Western blot analyses with the indicated antibodies. (C-D) HCT116 cells were infected with CHOP siRNA, ATF4 expression in HCT116 cells was determined by Western blot analyses after stimulation with the combination for 6 h. Data from three technical replicates. (* $p < 0.05$, ** $p < 0.01$).

Figure S5

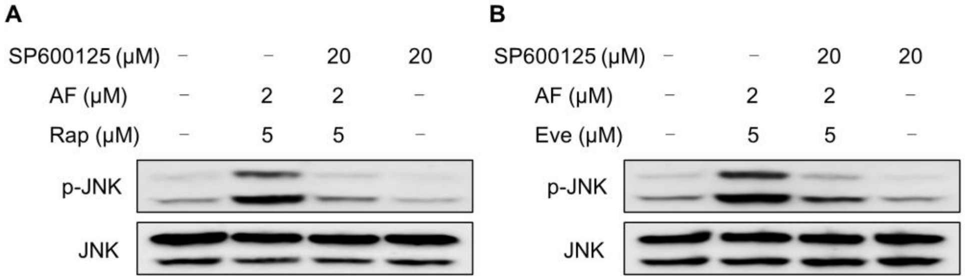

Figure S5. Pretreatment with SP600125 markedly reversed the phosphorylation of JNK induced by the combination of mTOR inhibitors and auranofin in SGC-7901. (A-B) SGC-7901 cells were pretreated with SP600125 (20 μM) for 2 h and cell lysates were blotted with the indicated antibodies after treated with auranofin and rapamycin combination for 12 h. (B) SGC-7901 cells were pretreated with SP600125 (20 μM) for 2 h and cell lysates were blotted with the indicated antibodies after treated with auranofin and everolimus combination for 12 h. Data from three technical replicates.

Figure S6

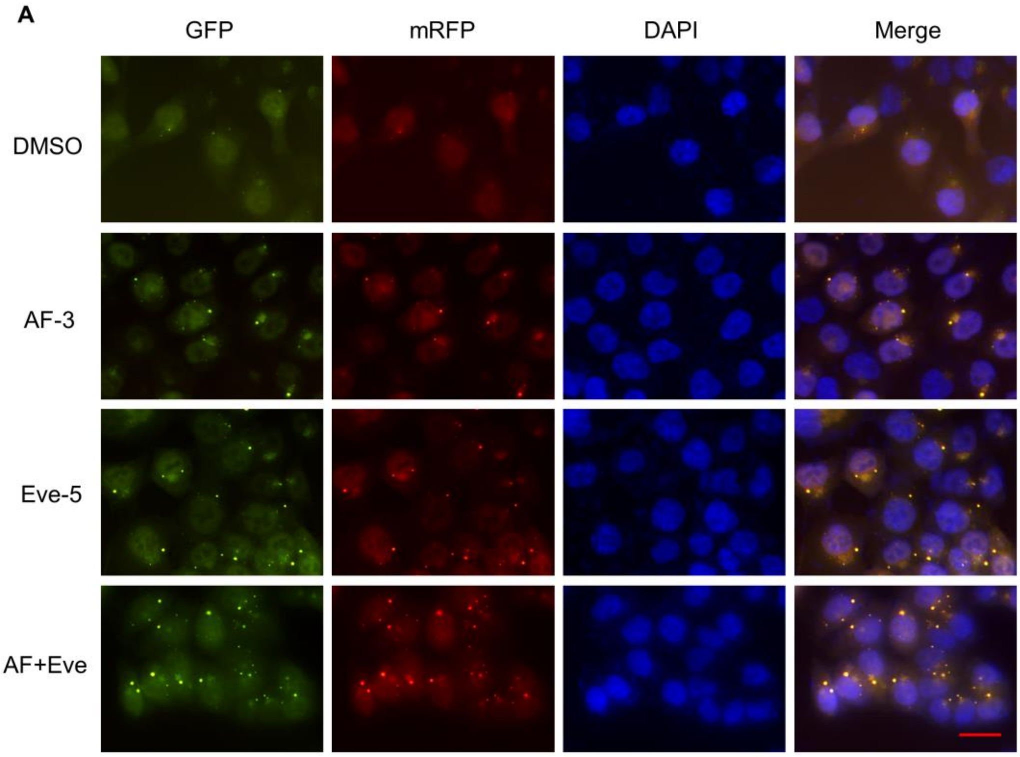

Figure S6. mTOR and TrxR inhibitor cooperate to induce autophagy. (A) The infected HCT116 cells were treated with auranofin (3 μM) or everolimus (5 μM) alone or their combination (3 μM auranofin and 5 μM everolimus) for 2 h and were then analyzed by fluorescence microscope. Scale bar = 25 μm. Data from three technical replicates.

Figure S7

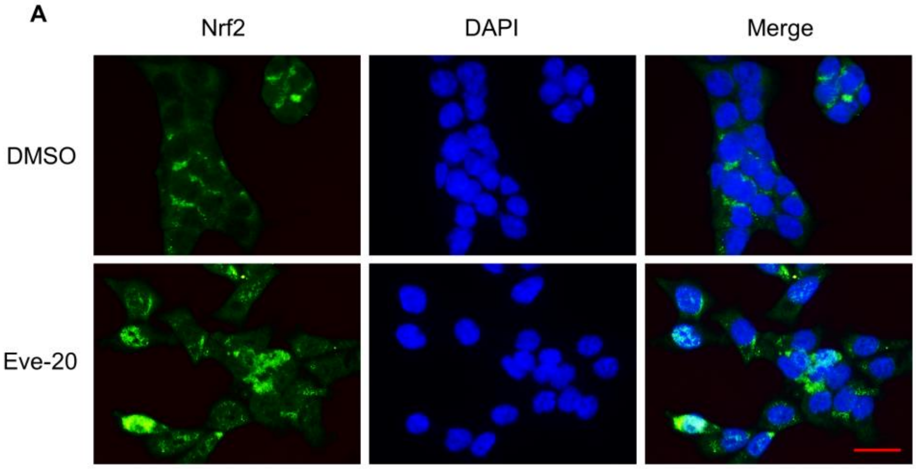

Figure S7. The expression of Nrf2 and nuclear Nrf2 was significantly increased in response to everolimus. (A) HCT116 cells after treatment with everolimus (20 μM) for 9 h were stained with Nrf2 and were then analyzed by fluorescence microscope. Scale bar = 25 μm. Data from three technical replicates.

Figure S8

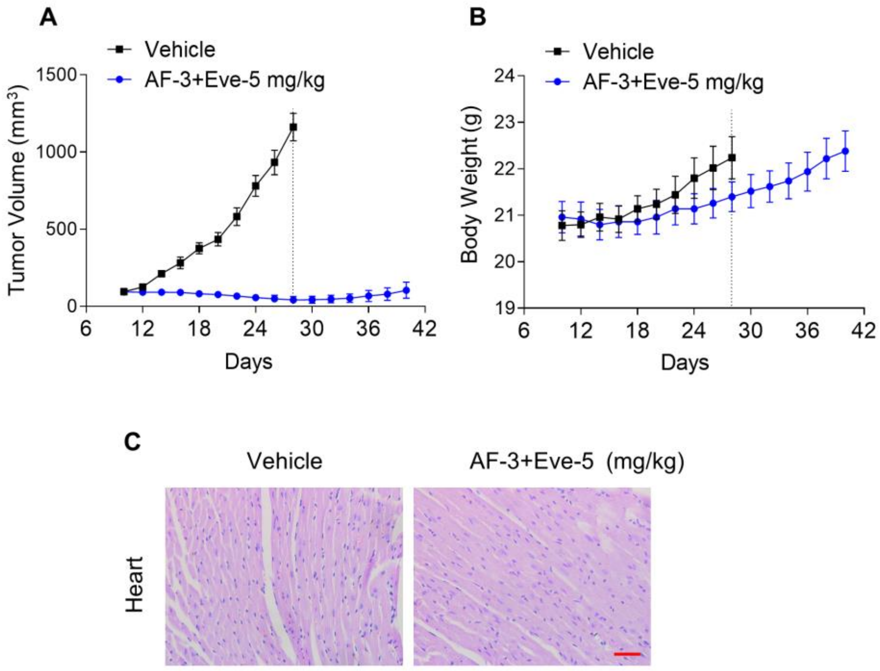

Figure S8. Everolimus and auranofin combination significantly inhibited tumor growth in nude mice. (A-B) Everolimus (5 mg/kg) and auranofin (3 mg/kg) combined treatment significantly decreased tumor volume of SGC-7901 human gastric cancer xenografts in nude mice, but did not significantly affect body weight of mice. The combination treatment was stopped on the 28th day. (C) HE staining of heart tissue. Scale bar = 75 μm.
